# Schistosomiase importée chez des migrants mineurs non accompagnés à Nice (France)

**DOI:** 10.48327/mtsi.v6i1.2026.659

**Published:** 2026-01-28

**Authors:** Frédéric VANDENBOS, Karine RISSO, Flore PETIT, Mai Ly DURANT, Philippe BABE, Pascal DELAUNAY, Loïc SIMON, Pierre MARTY, Christelle POMARES, Michel CARLES

**Affiliations:** 1 Centre de lutte antituberculeuse de Nice. Hôpital Pasteur, 30 Voie romaine, 06000 Nice, France; 2 Service des maladies infectieuses et tropicales. Hôpital Archet, 151, route Saint-Antoine de Ginestière 06202 Nice Cedex 3, France; 3 Service départemental de protection maternelle et infantile des Alpes-Maritimes. 147 boulevard du Mercantour, BP 3007, 06201 Nice Cedex 3, France; 4 Centre de permanence d’accès aux soins pédiatriques. Hôpital Lenval, 57 avenue de la Californie, 06200 Nice, France; 5 Service de parasitologie et de mycologie. Hôpital Archet, 151, route Saint-Antoine de Ginestière 06202 Nice Cedex 3, France

**Keywords:** Schistosomiase, Migrants mineurs non accompagnés, Dépistage, Afrique subsaharienne, France, Schistosomiasis, Unaccompanied minor migrants, Screening, Sub-Saharan Africa, France

## Abstract

**Introduction:**

La fréquence de la schistosomiase importée par les migrants mineurs non accompagnés (MNA) est mal connue en France.

**Méthodologie:**

Nous avons recherché, entre décembre 2023 et février 2024, une schistosomiase chez les MNA en plus du dépistage de la tuberculose dans notre Centre de lutte antituberculeuse des Alpes-Maritimes. Un dépistage sérologique (Western Blot) a été proposé à tous les MNA originaires d’Afrique subsaharienne (ASS). En cas de positivité, un examen parasitologique des urines et des selles a été proposé.

**Résultats:**

Au cours de ces trois mois, 187 MNA d’ASS ont été dépistés. La sérologie était positive pour 116 (62 %) d’entre eux. L’âge médian était de 16 ans et le sex-ratio (H/F) de 6,9. Des examens parasitologiques ont pu être réalisés pour 58 (50 %) des 116 MNA ayant une sérologie positive. Des œufs de schistosome ont été trouvés chez 24 d’entre eux. Il y avait des œufs de *Schistosoma mansoni* dans les selles de 14 MNA, des œufs de *S. haematobium* dans les urines de 9 MNA et la présence à la fois de *S. mansoni* et de *S. haematobium* chez 1 MNA. Cinq MNA présentaient une hématurie (avec *S. haematobium*) au moment de l’étude. La prévalence de la schistosomiase était d’au moins 12,5 % (24/187).

**Discussion/Conclusion:**

La schistosomiase est fréquente chez les MNA et les formes asymptomatiques sont les plus nombreuses.

## Abstract (en)

**Introduction:**

The prevalence of imported schistosomiasis among unaccompanied minor migrants (UMMs) in France is not well understood.

**Methodology:**

From December 2023 to February 2024, we screened UMMs for schistosomiasis, in addition to tuberculosis screening, at our Alpes-Maritimes Tuberculosis Control Center. We offered serological screening (Western Blot) to all UMMs from sub-Saharan Africa (SSA). If the result was positive, we offered a parasitological examination of urine and stool samples.

**Results:**

During these three months, we screened 187 UMMs from SSA. Serology was positive for 116 of them (62%). The median age was 16 years, and the sex ratio (male to female) was 6.9. Parasitological tests were performed on 58 (50%) of the 116 UMMs with positive serology results. Schistosome eggs were found in 24 of them. Fourteen UMMs had *Schistosoma mansoni* eggs in their stools, nine had *S. haematobium* eggs in their urine, and one had both *S. mansoni* and *S. haematobium*. Five UMMs had hematuria (due to *S. haematobium*) at the time of the study. The prevalence of schistosomiasis was at least 12.5% (24 out of 187).

**Discussion/Conclusion:**

Schistosomiasis is common among UMMs, with asymptomatic cases being the most prevalent.

## Introduction

La schistosomiase touche plus de 220 millions de personnes dans le monde dont plus de 90 % vivent en Afrique subsaharienne (ASS). Malgré la distribution massive de praziquantel aux enfants d’âge scolaire promue par l’Organisation mondiale de la Santé (OMS) vers le début de la décennie 2000-2010, la schistosomiase reste un problème majeur en Afrique de l’Ouest [4].

L’importance de la schistosomiase parmi les migrants mineurs non accompagnés (MNA) d’ASS est mal connue en France [5,6]. L’objectif de notre étude était d’appréhender ce risque dans une région où un des hôtes intermédiaires du genre *Bulinus* est présent, faisant courir le risque d’une implantation de la transmission de cette parasitose [2].

## Méthodologie

L’étude s’est déroulée dans le Centre de lutte antituberculeuse (CLAT) de Nice entre décembre 2023 et février 2024 (3 mois). En plus du dépistage de la tuberculose, nous avons proposé celui de la schistosomiase, par examen sérologique, à tous les MNA amenés dans notre centre. En cas de positivité, une recherche d’œufs parasitaires a été proposée dans les urines et les selles.

La séroprévalence était définie par le pourcentage de sérologies positives dans la population étudiée. La schistosomiase active était définie par la présence d’œufs de *Schistosoma* dans les excreta. Le test sérologique de la schistosomiase était réalisé par Western Blot conformément aux instructions du fabricant (IgG LDBIO Diagnostics®). La présence d’une bande de 22-24 kDa et/ou de 30-34 kDa était considérée comme positive.

L’examen microscopique des urines était réalisé après centrifugation. Les échantillons de selles étaient examinés au microscope après concentration et coloration dans un milieu iodésine (Para-selle Biosynex®, France) et Bailenger. Un seul prélèvement de selles était réalisé pour chaque jeune dans notre étude.

La comparaison des données qualitatives entre les pays d’origine divisés en trois groupes (Guinée, Côte d’Ivoire et autres) et entre les sexes était réalisée à l’aide du test du Chi^2^. Nous n’avons pas précisé les intervalles de confiance des prévalences de schistosomiase observées puisqu’il ne s’agit pas d’échantillonnage. Tous les tests étaient bilatéraux avec un seuil de significativité de 5 %. L’analyse statistique était réalisée à l’aide du logiciel Statistical Package for Social Sciences (SPSS, Carry; Inc. 22.0).

Le CLAT des Alpes-Maritimes dispose d’un enregistrement auprès de la Commission nationale de l’informatique et des libertés (CNIL) pour réaliser le recueil de données (fiche de registre n° 2023-EI-588). Les consultations étaient réalisées en présence d’une personne de confiance du jeune migrant (son éducateur). Lors de cette consultation, son consentement oral était demandé. Un traducteur était sollicité lorsque le jeune ne parlait pas le français.

## Résultats

**Figure 1:**
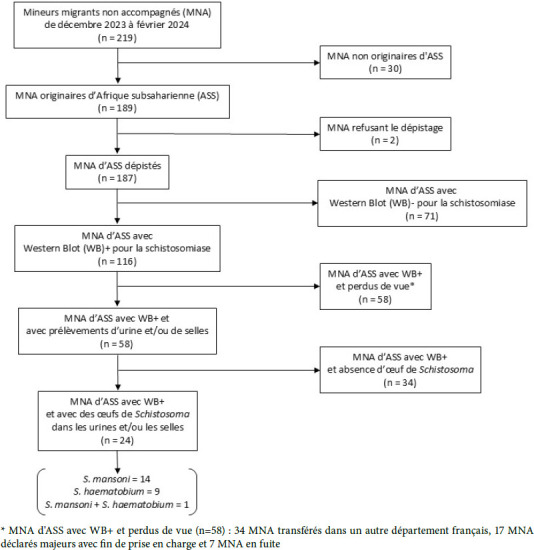
Schéma du dépistage de la schistosomiase chez les mineurs migrants non accompagnés (MMA) pendant les trois mois de l’étude

De décembre 2023 à février 2024, 187 MNA ont accepté le dépistage de la schistosomiase (Fig. 1). La plupart venait de Guinée (n = 85) ou de Côte d’Ivoire (n = 70). Les autres venaient de 10 pays différents (n = 32). L’âge médian déclaré était de 16 ans et le sex-ratio (H/F) de 6,9.

La sérologie de la schistosomiase était positive pour 116/187 MNA (62 %). La répartition des sérologies positives par pays était de 69 % (59/85) pour la Guinée, 57 % (40/70) pour la Côte d’Ivoire et 53 % (17/32) pour tous les autres pays : Mali (10/12), Cameroun (1/7), Burkina Faso (3/3), Gambie (1/3), Niger (0/2), Sénégal (0/1), Sierra Léone (0/1), Somalie (1/1), Sud Soudan (0/1), Tchad (1/1), sans différence significative entre les pays. Il existait une différence entre les sexes avec 32 % (8/25) de sérologies positives chez les jeunes femmes et 66 % (108/162) pour les jeunes hommes (p < 0,05). Parmi les 58 MNA ayant réalisé des analyses parasitologiques urinaires et/ou de selles, 24 présentaient des œufs de *Schistosoma*. On comptait 14 MNA avec *S. mansoni* dans les selles, 9 MNA avec *S. haematobium* dans les urines et un jeune avec à la fois *S. mansoni* dans les selles et *S. haematobium* dans les urines (Fig. 1). Parmi les 24 MNA avec une schistosomiase active, 5 présentaient une hématurie (*S. haematobium*). La prévalence de la schistosomiase active était ainsi d’au moins 12,5 % (24/187).

Tous les MNA présentant une sérologie positive ayant rapporté leurs prélèvements urinaires et/ou fécaux ont été traités par praziquantel à 40 mg/kg.

## Discussion

Dans notre population de MNA d’ASS, la séroprévalence de la schistosomiase était de 62 %, la prévalence de la schistosomiase active était d’au moins 12,5 %.

La fréquence de la schistosomiase est imparfaitement connue en France chez les jeunes migrants d’ASS. La séroprévalence dans une étude parisienne concernant des enfants était de 25,5 % [5]. Nos résultats sont plus de deux fois supérieurs. Cependant, les MNA de cette étude ne représentaient que 42,1 % de la population pédiatrique étudiée. L’étude de Naccache *et al.* concernait quant à elle 48 garçons d’Afrique de l’Ouest présentant une schistosomiase uro-génitale [6]. Ces enfants présentaient les mêmes caractéristiques épidémiologiques que dans notre étude. Cependant, ils étaient pour la plupart symptomatiques, raison pour laquelle ils étaient inclus. Dans notre étude, le dépistage était systématique et mettait en évidence de nombreux cas de schistosomiase asymptomatique.

Le dépistage de la schistosomiase en France était réservé, jusqu’à présent, aux seuls patients symptomatiques [3,6]. Récemment, la Société de pathologie infectieuse de langue française (SPILF) a émis des recommandations pour un dépistage systématique, au moins sérologique, chez les migrants originaires d’une zone d’endémie, mais ces recommandations ne sont pas contraignantes [7]. La recherche d’œufs de *Schistosoma* dans les excreta étant fastidieuse et chronophage, nous avons cessé cet examen, même s’il est pourtant l’examen de référence pour diagnostiquer une schistosomiase active [1]. Cependant, pour faire le diagnostic et instaurer un traitement, la meilleure solution semble être la sérologie comme préconisé par la SPILF [7].

Notre travail présente un certain nombre de biais. D’une part, nous avons utilisé comme test sérologique le Western Blot. En France le test recommandé en première intention est plutôt un test ELISA et/ou IHA, suivi du Western Blot de confirmation en cas de positivité [7]. Le test Western Blot a l’avantage d’être plus spécifique [1]. D’autre part, la prévalence de la schistosomiase active dans notre population est sans doute sous-estimée car même si c’est la méthode de référence, la recherche d’œufs de *Schistosoma* dans les excreta est peu sensible [1]. De plus, cette recherche a été réalisée sur une seule selle au lieu des trois recommandées [1]. Enfin, 50 % de nos MNA ont été perdus de vue ce qui sous-estime d’autant la prévalence.

Les nombreux perdus de vue de notre étude soulèvent deux questions. D’une part, le risque d’implantation de la schistosomiase dans les plans d’eau douce du pourtour méditerranéen français où le mollusque hôte intermédiaire (*Bulinus truncatus*) est présent. L’exemple corse est, à ce titre, exemplaire [2]. D’autre part, non traitée, cette parasitose est à l’origine d’une morbidité importante pour le porteur, en particulier pour les formes urogénitales [6].

## Conclusion

En conclusion, la schistosomiase est fréquente chez les jeunes migrants originaires d’ASS. Un dépistage systématique est pertinent.

## Remerciements

Nous remercions tous les foyers des Alpes-Maritimes prenant en charge les jeunes migrants mineurs non accompagnés, ainsi que tous les éducateurs qui nous ont aidés à récolter les prélèvements urinaires et de selles.

## Financement

Cette étude n’a reçu aucune subvention spécifique d’un organisme de financement des secteurs public, commercial ou à but non lucratif.

## Contributions des auteurs et autrices

FV et KR ont contribué de manière égale à la conceptualisation, à la collecte de données, à la rédaction de la version originale, à la révision et à l’édition.

FV a effectué l’analyse statistique et contribué à la méthodologie.

FP, PB, PD et LS ont contribué à la conceptualisation, à la validation, à la révision et à l’édition. MLD, PM, CP et MC ont supervisé et validé l’étude.

## Déclaration de liens d’intérêts

Aucun intérêt financier connu ni aucune relation personnelle qui aurait pu sembler influencer le travail rapporté dans cet article n’a été déclaré.
