# Supplementary material for: Imported Schistosomiasis among unaccompanied minor migrants in Nice (France)
Source: Med Trop Sante Int. 2026 Jan 28;6(1):mtsi.v6i1.2026.803. [Article in French] doi: 10.48327/mtsi.v6i1.2026.659 (PMC13216897; doi:10.48327/mtsi.v6i1.2026.659)
Supplement: Related texts

# Research article: Imported Schistosomiasis among unaccompanied minor migrants in Nice (France)

## Introduction

Schistosomiasis affects over 220 million people worldwide, most of whom live in sub-Saharan Africa (SSA). Despite the World Health Organization’s (WHO) promotion of the mass distribution of praziquantel to school-aged children in the early 2000s, schistosomiasis remains a major problem in West Africa [4].

The prevalence of schistosomiasis among unaccompanied migrant minors (UMMs) from SSA is not well understood in France [5,6]. Our study aimed to assess this risk in a region where one of the intermediate host genus *Bulinus* is present and could lead to the establishment of the transmission of this parasitic disease [2].

## Methodology

The study was conducted at the Tuberculosis Control Center (TCC) in Nice during 3 months from December 2023 to February 2024. In addition to tuberculosis screening, we offered schistosomiasis screening by serological examination to all UMMs brought to our center. In the event of a positive result, we offered a search for parasitic eggs in urine and stool samples.

Seroprevalence was defined as the percentage of positive serology results in the study population. Active schistosomiasis was defined as the presence of *Schistosoma* eggs in excreta.

We performed the serological test for schistosomiasis by Western Blot according to the manufacturer’s instructions (IgG, LDBIO Diagnostics®). The presence of a 22-24 kDa and/or a 30-34kDa band was considered positive.

A microscopic examination of the urine was performed after centrifugation. Stool samples were examined under a microscope after being concentrated and stained in an iodine medium (Para-Selle, Biosynex®, France) and using the Bailenger method. Only one stool sample was collected from each child in our study.

We compared qualitative data between countries of origin, which we divided into three groups (Guinea, Côte d’Ivoire, and others), and between sexes using the chi-squared test. We did not specify confidence intervals for the observed prevalence of schistosomiasis because they were not samples. All tests were two-tailed with a significance threshold of 5%. Statistical analysis was performed using the Statistical Package for the Social Sciences (SPSS, Inc., version 22.0). The TCC in the Alpes-Maritimes region is registered with the *Commission Nationale de l’Informatique et des Libertés* (National Commission on Informatics and Liberty) to collect data (registration number 2023-EI-588). The consultations were conducted in the presence of a trusted adult (educator) for the young migrant. During the consultation, verbal consent was requested. A translator was called in when the young person did not speak French.

## Results

From December 2023 to February 2024, 187 UMMs agreed to be screened for schistosomiasis (Fig. 1). Most came from Guinea (n = 85) or Côte d’Ivoire (n = 70). The rest came from ten different countries (n = 32). The median reported age was 16 years, and the sex ratio (M/F) was 6.9.

Schistosomiasis serology was positive for 116 out of 187 UMMs (62%). The serology results were distributed as follows by country: 69% (59/85) in Guinea, 57% (40/70) in Côte d’Ivoire, and 53% (17/32) in all other countries: Mali (10/12), Cameroon (1/7), Burkina Faso (3/3), Gambia (1/3), Niger (0/2), Senegal (0/1), Sierra Leone (0/1), Somalia (1/1), South Sudan (0/1), and Chad (1/1). There was no significant difference between countries. However, there was a difference between the sexes: 32% (8/25) of serology tests were positive in young women, compared to 66% (108/162) in young men (p < 0.05).

Of the 58 UMMs who underwent parasitological analysis of urine and/or stool samples, 24 had *Schistosoma* eggs. Fourteen UMMs had *S. mansoni* in their stools, nine had *S. haematobium* in their urine, and one had both *S. mansoni* in their stools and *S. haematobium* in their urine (Fig. 1). Of the 24 UMMs with active schistosomiasis, five had hematuria (*S. haematobium*). The prevalence of active schistosomiasis was at least 12.5% (24 out of 187).

All UMMs with positive serology who provided urine and/or stool samples were treated with praziquantel at 40 mg/kg.

**Figure 1:** Diagram of schistosomiasis screening among unaccompanied migrant minors (UMMs) during the three months of the study

## Discussion

In our population of UMMs from SSA, the seroprevalence of schistosomiasis was 62%, and the prevalence of active schistosomiasis was at least 12.5%.

The prevalence of schistosomiasis among young migrants from SSA in France is not well known. A Parisian study of children found a seroprevalence of 25.5% [5]. Our results are more than twice as high. However, UMMs represented only 42.1% of the pediatric population in this study. Naccache *et al.*’s study involved 48 boys from West Africa with urogenital schistosomiasis [6]. These children had the same epidemiological characteristics as those in our study. However, most of them were symptomatic, which is why they were included in the study. In our study, systematic screening revealed many cases of asymptomatic schistosomiasis.

Until now, schistosomiasis screening in France has been reserved only for symptomatic patients [3,6]. Recently, the *Société de pathologie infectieuse de langue française* (SPILF) issued recommendations for systematic screening, at least serological, of migrants from endemic areas. However, these recommendations are not binding [7].

Since searching for *Schistosoma* eggs in feces is tedious and time-consuming, we discontinued this test even though it is the gold standard for diagnosing active schistosomiasis [1]. The best solution for making a diagnosis and initiating treatment seems to be serology, as recommended by the SPILF [7].

Our work has a number of biases. First, we used Western Blot as the serological test. In France, the recommended first-line tests are ELISA and/ or IHA, followed by Western Blot for confirmation in cases of positivity [7]. The Western Blot test has the advantage of being more specific [1]. Second, the prevalence of active schistosomiasis in our population is undoubtedly underestimated because searching for *Schistosoma* eggs in excreta, even though it is the reference method, is not very sensitive [1]. Additionally, this search was carried out using a single stool sample instead of the recommended three samples [1]. Finally, 50% of our UMM patients were lost to follow-up, which further underestimates the prevalence. This large number of patients lost to follow-up in our study raises two questions. First, there is a risk that schistosomiasis will become established in the freshwater bodies around the French Mediterranean coast, where the intermediate host mollusk (*Bulinus truncatus*) is present. Corsica is an illustrative example in this regard [2]. Second, if left untreated, this parasitic disease causes significant morbidity for the carrier, particularly in its urogenital forms [6].

## Conclusion

In conclusion, schistosomiasis is frequent among young migrants from SSA. Systematic screening is appropriate.

## Acknowledgments

We would like to thank all the shelters in the Alpes-Maritimes region that take care of unaccompanied minor migrants, as well as all the educators who helped us collect urine and stool samples.

## Funding

This study did not receive funding from any public, commercial, or nonprofit agency.

## Author’s contributions

FV and KR contributed equally to conceptualization, data collection, writing the original version, revising, and editing.

FV performed the statistical analysis and contributed to the methodology.

FP, PB, PD, and LS contributed to the conceptualization, validation, revision, and editing.

MLD, PM, CP, and MC supervised and validated the study.

## Conflicts of interest

No financial or personal conflicts of interest that could influence the work reported in this article were declared.
